# Opinion: Research Progress of Surgical Treatment of Osteoarthritis

**DOI:** 10.3389/fsurg.2022.922091

**Published:** 2022-05-16

**Authors:** Peng Zheng, Min Li, Keren Cao, Yang Liu, Yunrun Liu

**Affiliations:** ^1^ Jinan Institute for Food and Drug Control, Jinan, China; ^2^ Hong Kong Baptist University, Kowloon, Hong Kong, China

**Keywords:** surgical treatment, osteoarthritis, artificial joint replacement, arthroscopic debridement, minimally invasive surgery (MIS)

## Introduction

Osteoarthritis (OA) is one of the most common joint diseases, mainly due to the degeneration of articular cartilage and the abnormal biosynthesis of proteoglycan in articular cartilage, which seriously affects the quality of life of patients. At present, the incidence of osteoarthritis is mainly concentrated in middle-aged and elderly people over 40 years old, with a prevalence rate of 17.0%. However, there are also studies showing that the incidence still exists in children and infants, but it is more related to genes and environment. In the next few decades, the prevalence of OA will gradually increase, which will seriously affect people's health and quality of life, and bring a huge burden to China's national economic and social development (1).

In addition to affecting physical health, OA can also affect mental health. Studies have shown that patients with OA have a higher probability of depressive symptoms than healthy groups (2). Another study found a strong relationship between OA and memory loss (3). At the same time, OA can increase the risk of cardiovascular disease (4–6).

**Figure 1:**
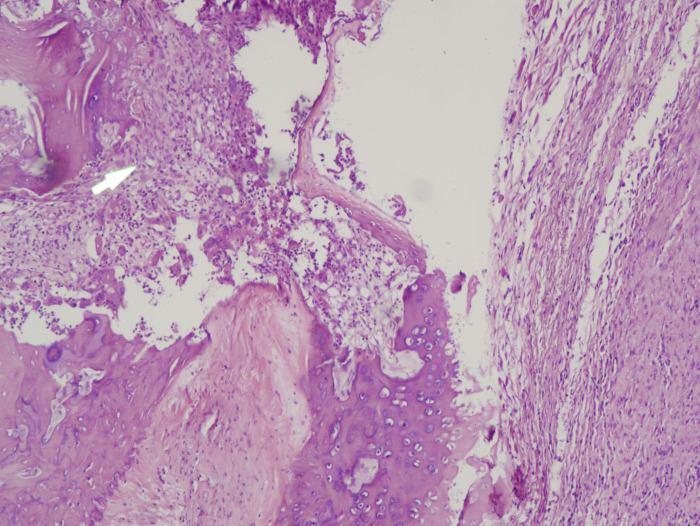
Rat osteoarthritis(Cartilage cell hypertrophy and differentiation).

The risk factors of OA are diverse, including heredity, aging, obesity and so on. In the early stage of OA, the cartilage surface is still intact. The lesions first manifest as molecular changes in the extracellular matrix (7), Figure 1: Rat osteoarthritis(Cartilage cell hypertrophy and differentiation); accompanied by chondrocyte hypertrophy and differentiation, marker expression, destruction of cartilage integrity, and subsequent apoptosis of articular chondrocytes (8). Relevant studies have found that cytokines are closely related to the pathogenesis of OA. In patients with OA, cartilage matrix homeostasis is destroyed by pro-inflammatory cytokines and chemokines (IL-1, IL-6, and IL-8), which stimulate macrophages and chondrocytes to co-produce proteases, nitric oxide (no) and eicosanoids, etc, which participate in the process of catabolism and induce apoptosis (9). which in turn induces synthesis of MMPs (MMP-1, MMP-3, and MMP-13) through amplification of pro-inflammatory cytokines (such as TNF-α, IL-6 and IL-8), increase the destruction of articular cartilage cells (10).

At present, according to the progress of the disease, its treatment methods are mainly divided into three categories. Firstly, basic treatment mainly focuses on acupuncture, moxibustion, massage and exercise in traditional Chinese medicine, mainly for patients with mild symptoms; Secondly, drug treatment, mainly non steroidal anti-inflammatory drugs, analgesics, intra-articular injection drugs, etc; Thirdly, surgical treatment is mainly for patients with advanced OA, such as articular cartilage repair, arthroscopic cleaning, artificial joint replacement and so on.

## Main Surgical Treatment of Osteoarthritis

### Joint Debridement

Joint debridement is a basic surgical operation. The early treatment of joint diseases plays a decisive role in wound healing and the recovery of the function and shape of injured tissues, which should be paid attention to. In clinic, joint debridement is often combined with artificial prosthesis surgery. In order to prevent the formation of sinus tract in joint surgery, joint debridement is often used to keep the prosthesis, and the patient is debrided at the right operation time, so as to prevent the fever caused by infection from being difficult to cure and hinder the formation of the final sinus tract. In most cases, debridement can effectively remove the dead and inflammatory tissues around the joints of patients, and can significantly reduce the recurrence rate of patients with low-grade infection.

### Arthroscopic Debridement

For mild to moderate arthritis, joint debridement is recommended, which can effectively remove intra-articular effusion and improve joint function. However, in view of the older age of OA patients and the fact that arthroscopic debridement can not completely repair bone joints and damaged cartilage tissue, some patients treated with arthroscopic debridement alone are not effective. And in the current surgery, the patient will be placed in the supine position, and the waist will be anesthetized, and the surgical site will be disinfected with a disinfectant spread towel. Most of the surgical methods are to take the anteromedial approach of the knee joint to operate on the patient, and then the operating instruments will be placed in turn, and the anteromedial approach will be used to place the light source.

The researchers found that ultrasonic conduction percutaneous local drug penetration has the advantages of high local drug concentration and avoidance of liver first-pass effect and is widely used in clinical practice. Therefore, after arthroscopic debridement, topical ultrasound percutaneous administration can achieve better results, can significantly relieve the patient's knee joint pain, swelling and other symptoms, and improve knee joint function. Its mechanism of action may be related to this combination therapy. The expression levels of BGP, OPG, and RANKL, which are biochemical indicators of benign regulation of bone metabolism, were significantly down-regulated in vivo. However, arthroscopy technology can not fully meet the needs of patients, mainly for patients with moderate osteoarthritis. Severe patients eventually need artificial joint replacement to solve the end-stage joint degeneration.

### Artificial Joint Replacement

Artificial joint replacement is the most effective surgical method for the treatment of end-stage degenerative joint diseases and other joint diseases. It can improve the joint mobility and relieve patients’ joint pain symptoms in the short term after surgery, so as to meet the requirements of high quality of life for patients. During the operation, the surgeon will adjust according to the patient's operation position, such as knee joint, hip joint, ankle joint, etc. will perform lower limb operation in supine position, while shoulder joint, elbow joint, etc. will perform upper limb operation in beach chair position (a supine position in which the upper body is higher than the operating table plane); After that, the doctor will cut the tissue of the patient's diseased position to expose the joints at the surgical site; Remove the joint replacement part; Implant artificial prosthesis; Confirm whether the reduction and fixation are satisfactory; Rinse the incision, and finally sew and bandage.

As stated in the manuscript before, the biggest problem in artificial joint replacement is the aseptic loosening of the prosthetic joint, which is the main complication affecting the life of the prosthesis. Complications caused by prosthesis infection will greatly reduce the recovery rate of patients, and there is a higher possibility that patients can't bear the discomfort and pain caused by complications, and even take their lives. It seriously hinders the popularization and application of artificial joints and people's confidence in joint replacement surgery. Prosthetic joint revision surgery is much more difficult than primary joint replacement surgery. In the operation, there will be a bigger problem on the surgical surface, and there is a great risk of infection in debridement, and the postoperative recurrence rate is extremely high. And revision surgery is more traumatic, has a high risk of death during the operation, and has poor postoperative joint function recovery, which often brings huge physical and economic burdens to patients.

## Discussion

The development of materials science has become a key research field in bone and joint development. And the most important mechanism of prosthesis is the absorption and dissolution of bone around the prosthesis induced by wear particles. Therefore, the first step is to explore the mechanism of osteolysis from the biological point of view and find the key biological factors, so as to prevent the dissolution of bone tissue around the prosthesis; Secondly, focus on the prosthesis materials, to create more wear-resistance and more compatible with human tissue prosthesis materials. 3D printing technology can print more personalized joint materials, with better wedge fit, which is more conducive to the recovery of patients.

In recent years, minimally invasive surgery has gradually become the treatment trend of knee OA because of its advantages of small trauma and rapid recovery. Minimally invasive technology can reduce patients’ pain, reduce the amount of bleeding and have better postoperative activity function, but it is difficult to ensure the accuracy of prosthesis position. Recently, with the application of computer navigation and robot technology in treatment, the defect of inaccurate positioning of minimally invasive technology has been made up. Computer navigation can accurately calculate the amount of osteotomy and guide the position and angle of prosthesis placement. Compared with the traditional method, it has the advantages of high precision of prosthesis implantation, good recovery of lower limb force line and good recovery of postoperative knee function (11). Moreover, the update of technology has made the age problem of patients gradually disappear. In the future, no matter how old or old the patients are, there will be a chance to be cured. And the development of interdisciplinary integration will inevitably bring more convenience and advantages to the surgical treatment of bone and joint patients.

## References

[1] Liu Y Zhang H Liang N Fan W Li J Huang Z Prevalence and associated factors of knee osteoarthritis in a rural Chinese adult population: an epidemiological survey. BMC Public Health. (2015) 16:94. 10.1186/s12889-016-2782-x PMC4736305 26830813

[2] Veronese N Stubbs B Solmi M Smith TO Noale M Cooper C Association between lower limb osteoarthritis and incidence of depressive symptoms: data from the osteoarthritis initiative. Age Ageing. (2016) 46(3):470–6. 10.1093/ageing/afw216 27932358

[3] Innes KE Sambamoorthi U. The association of perceived memory loss with osteoarthritis and related joint pain in a large appalachian population. Pain Med. (2017) 19(7):7. 10.1093/pm/pnx107 PMC6279269 28525629

[4] Schieir O Tosevski C Glazier RH Hogg-Johnson S Badley EM. Incident myocardial infarction associated with major types of arthritis in the general population: a systematic review and meta-analysis. Ann Rheum Dis. (2017) 76(8):1396–404. 10.1136/annrheumdis-2016-210275 28219882

[5] Chung WS Lin HH Ho FM Lai CL Chao CL. Risks of acute coronary syndrome in patients with osteoarthritis: a nationwide population-based cohort study. Clin Rheumatol. (2016) 35(11):2807–13. 10.1007/s10067-016-3391-x 27585925

[6] Courties A Sellam J Maheu E Cadet C Barthe Y Carrat F Coronary heart disease is associated with a worse clinical outcome of hand osteoarthritis: a cross-sectional and longitudinal study. Rmd Open. (2017) 3(1):e000344. 10.1136/rmdopen-2016-000344 28243467 PMC5318565

[7] Goldring MB Goldring SR. Articular cartilage and subchondral bone in the pathogenesis of osteoarthritis. Ann N Y Acad Sci. (2010) 1192:1192. 10.1111/j.1749-6632.2009.05240.x 20392241

[8] Mort JS Billington CJ. Articular cartilage and changes in Arthritis: matrix degradation. Arthritis Res. (2001) 3(6):337–41. 10.1186/ar325 11714387 PMC128908

[9] Abramson SB Attur M. Developments in the scientific understanding of osteoarthritis. Arthritis Res Ther. (2009) 11(3):227. 10.1186/ar2655 19519925 PMC2714096

[10] Hoff P Buttgereit F Burmester G-R Jakstadt M Gaber T Andreas K Osteoarthritis synovial fluid activates pro-inflammatory cytokines in primary human chondrocytes. Int Orthop. (2013) 37(1):145–51. 10.1007/s00264-012-1724-1 23212731 PMC3532646

[11] Horikawa A Miyakoshi N Shimada Y Kodama H. Comparison of clinical outcomes between total knee arthroplasty and unicompartmental knee arthroplasty for osteoarthritis of the knee: a retrospective analysis of preoperative and postoperative results. J Orthop Surg Res. (2015) 10:168. 10.1186/s13018-015-0309-2 26510773 PMC4625455

